# A Pilot Study of an Interprofessional Program Involving Dental, Medical, Nursing, and Pharmacy Students

**DOI:** 10.3389/fpubh.2020.602957

**Published:** 2020-12-10

**Authors:** Maryam Tabrizi, Wei-Chen Lee

**Affiliations:** ^1^General Practice and Dental Public Health, School of Dentistry, The University of Texas Health Science Center at Houston, Houston, TX, United States; ^2^Office of Health Policy and Legislative Affairs, The University of Texas Medical Branch, Galveston, TX, United States

**Keywords:** oral health, interprofessional education, collaboration, age-friendly, public health

## Abstract

**Objective:** The silent epidemic of oral diseases disproportionately affects disadvantaged communities, especially the elderly who have complex needs for healthcare. This study was to evaluate a pilot oral health interprofessional program that provided hands-on experiences for students across four disciplines: dentistry, medicine, nursing, and pharmacy.

**Methods:** The 8-weeks program was built on four pedagogical principles: care, critical thinking, communication, and collaboration coupled with the 4Ms model: what matters, medication, mentation, and mobility. The curriculum contained four scenarios of a dental complication in an elderly: Alzheimer's Disease, oral cancer, Parkinson's Disease, and stroke. A mixed-methods approach was used to evaluate this pilot program.

**Results:** The average score of knowledge and attitude has increased from 2.94 to 4.39 (*p* < 0.05) on a 5-point Likert scale. The qualitative responses also showed that students became more confident in practicing within the Age-Friendly health system.

**Discussion:** By the end of the program, all students recognized the significance of the interprofessional program to improve their knowledge and skills to work with professionals across disciplines. Two key features that contributed to the success of the program were (1) an interprofessional education that increased students' awareness of other types of services and (2) four scenarios that allowed students to solve the case and gain hands-on experience.

**Conclusion:** An interprofessional education may equip students with competence to address the health of geriatric patients. Materials used in this study could be shared and adapted to prepare learners for other scenarios that require interprofessional team practice.

## Introduction

The population of the United States is aging and the Americans older than 65 are expected to account for 23 percent of total Americans by 2060 (1). Adults 65 years of age and older represent the most rapidly increasing demographic, particularly those aged 85 and older. The prevalence of chronic conditions associated with aging is also increasing, including cavities due to dry mouth, heart disease, cognitive decline, arthritis conditions, and a decline in cellular homeostasis during aging (1–4). Meanwhile, many chronic conditions such as heart disease negatively impact oral health (5). In response to this demographic trend, there is a high demand for a variety of healthcare providers including dentists who can work together to address the complex health needs of this population and achieve the best clinical outcomes (4).

The scope of practice has expanded for healthcare professionals (6, 7). Practicing dentistry is no longer limited to treating oral cavities, as dentists are “oral health physicians” who must be knowledgeable of related conditions and manage their patients appropriately. Similarly, physicians and those in all other healthcare disciplines should understand the impact of oral health on individual well-being and quality of life (7, 8). To facilitate collaborations among healthcare professionals, many studies indicate that creating an interactive educational platform may prepare healthcare students to develop a collaborative mindset (9–11). Sharing care across disciplines within an Age-Friendly health system is particularly essential in treating the aging population needing special care for underlying health conditions (12, 13).

Despite the fact that Interprofessional Education (IPE) has been widely employed since the 1960's, the American Dental Education Association did not establish its own IPE Collaborative until 2011 (14). IPE with medical (90%), pharmacy (76%), and nursing (62%) schools are common in dental schools, but only 12.5% of IPE programs combine all four disciplines into a care team (14, 15). Additionally, health systems are less likely to streamline dental services as a part of a care plan due to traditionally separate billing methods and practice sites (16). To close the gap between these four disciplines (medicine, nursing, pharmacy, and dentistry), we initiated an 8-weeks program in 2019 integrating the Interprofessional Education with the Interprofessional Collaboration Care (IPE-IPC) approaches (17, 18). The pilot program sought to train healthcare students to care for geriatric populations under the Age-Friendly system's 4Ms framework—what matters, medication, mentation, and mobility—while taking patients' oral issues into account (12). Namely, students have to adapt their practices not only to an interdisciplinary environment but also within an Age-Friendly setting.

The study aimed to describe the process of piloting an 8-weeks geriatric educational program and evaluate the effectiveness of the program in terms of students' knowledge and experiences. This is one of the first IPE-IPC programs to create an interactive environment for students from four disciplines (medicine, nursing, pharmacy, and dentistry). Students who enrolled in this program took part in sharing care to achieve the best health outcomes and improve the quality of life of frail older adults. In this study, we hypothesized that attending a clinically relevant IPE-IPC program will reinforce preclinical students' didactic knowledge and decrease anxiety when working in a real clinical situation. After attending this program, non-dental students were expected to be able to recognize the importance of oral health and dental students were expected to treat geriatric patients with the 4Ms model in mind. Also, we expected that all the students would acknowledge the value of interprofessional practice and gain confidence in collaborating with professionals across disciplines.

## Pedagogical Framework

The program was built on a team-based pedagogical model where students from four disciplines (dentistry, medicine, nursing, and pharmacy) work together to solve the health issues of standardized patients (17, 19). To be more specific, the pedagogical principles used in this program were care, critical thinking, communication, and collaboration. Care and critical thinking were two personal competences while communication and collaboration were two interpersonal competences. Table 1 demonstrated the individual roles and responsibilities in a care team. Students were encouraged to appreciate alternative views, foster a cohesive vision, and develop a case-based, patient-centered treatment plan addressing all aspects of the patient's conditions at the end of the class. The program's faculty and facilitators also provided feedback to students in each debriefing session so that students could improve their team's communication in the next class.

**Table 1:** Individual roles and responsibilities in an interprofessional team.

**Disciplines/scenarios**	**Dentistry**	**Medicine**	**Nursing**	**Pharmacy**
ALZ	Identify dental caries. Offer providing care and prevention. Care givers and family members are always to be involved. Particularly as the cognitive level declining.	In both office visits and hospital setting Assessment of oral cavity during initial exam to recognize abnormalities if any. Ask patients if they have problems in their mouth, if they can response, or discuss the situation with care givers or family members	Assessment of oral cavity during initial exam to recognize abnormalities if any. Ask patients if they have problems in their mouth. Assist them with routine daily brushing and rinsing, if they can response, or discuss the situation with care givers or family members	Remind individuals when medications may cause dry-mouth, suggest; Fluoridated alcohol-free mouth wash moisturizing mouth rinses The necessity of routine brushing and rinsing. if they are able to comprehend and respond, or discuss the situation with care givers or family members
OC	Identify oral erythroplakia	The same for all conditions	The same for all conditions	The same for all conditions
PKD	Identify advanced periodontal disease	The same for all conditions	The same for all conditions	The same for all conditions
STK	Identify abscessed tooth	The same for all conditions	The same for all conditions	The same for all conditions

*ALZ, Alzheimer's disease; OC, oral cancer; PKD, Parkinson's disease; STK, stroke*.

Next, we designed the educational activities by applying the 4Ms concept to treating four chronic conditions: Alzheimer's disease (ALZ), oral cancer (OC), Parkinson's disease (PKD), and stroke (STK). Faculty speakers were specialist experts in oral lesions, especially cancerous lesions and the periodontal diseases from which aging populations usually suffer. Every 2 weeks, faculty talked about an oral complication of a geriatric patient due to one of four chronic conditions. Students learned what matters the most to patients when they have oral issues. They also learned the importance of taking a comprehensive medication history and learned each drug's side effects and interactions with different chronic conditions. For different cases, students acquired knowledge of anti-coagulants, calcium channel blockers, or antipsychotic medications. Following that, students learned to acknowledge that caring for patients with mentation issues and cognitive decline involves much more skill for better care. In particular, managing patients with ALZ requires clinical skills as well as communication among providers to keep patients safe and achieve better clinical outcomes. Finally, students learned to recognize that mobility is a key contributing factor; they needed to ensure that older adults move safely every day to maintain function and do what matters. The details of each standardized patient case were as follows:

Scenario 1: STK patient is in a long-term care facility with an abscessed tooth, mild speech impairment, swallowing difficulties, osteoporosis, and overall muscular weakness in the right extremities.
Scenario 2: ALZ patient in a nursing home with severe recurrent caries under an old bridge, causing cellulitis. The patient also has a history of total knee replacement 15 years ago and breast cancer 5 years ago.
Scenario 3: PKD patient with advanced periodontal disease, missing a three-unit bridge on the lower left side of the mouth. The patient stops eating, due to mouth pain, but is not able to express the pain.
Scenario 4: Patient with squamous cell carcinoma appears as an Oral Erythroplakia lesion of the right side of the tongue. The patient also has been diagnosed as HIV-positive and with chronic renal failure.

## Learning Environment

The pilot study was designed to evaluate an IPE/IPC program that all activities were presented in a clinical setting. Students were encouraged to form a health team, practice share care and open communications with one another, and achieve the best practice and clinical outcomes. By using a mixed-methods approach, the study evaluated students' knowledge and experiences before and after attending this pilot IPE program. The following subsections provide more details on the content of this IPE-IPC program, the procedures of data collection, and the analysis plan.

### Program Objectives and Institutional Support

University of Texas Health Science Center at Houston was interested in setting up a program for students across health disciplines to experience IPE leading to IPC. An IPE-IPC program was expected to offer students an experience that would increase their knowledge about how oral health is connected to overall health, enabling them to recognize that shared care involves treating a patient as a whole person. Students were also expected to experience how a team of knowledgeable providers achieves the best clinical outcome together by employing mutual respect, active listening, and clear communication.

### Recruitment of Participants and Assignment of Groups

The pilot program took place for 8 weeks from February 14, 2019 to April 4, 2019. The pilot program was not yet a central curriculum in any of four disciplines: dentistry, medicine, nursing, and pharmacy. Consequently, volunteer students attended the program after their normal class time. Over 8 weeks, students met at the dental school on Thursday evenings from 5:30 to 7:00 pm for 90 min. Recruiting participants and analyzing our collected data required an additional 10 weeks, so the overall study period was from January 2, 2019 to May 14, 2019.

Students were recruited through geriatric faculty at four schools. The program team emailed a flier to faculty with information on the program's aims, eligibility to take part, time, and location. The program in total recruited 13 students in their 3rd or 4th year from these four schools: the medicine, nursing, and dentistry schools from University of Texas Health Science Center at Houston and the College of Pharmacy from Texas A&M University. Our intent was to recruit four volunteer students from each school, but we were able to recruit only one student from the College of Pharmacy. Following that, we divided the 13 students into four groups. Each group consisted of one student from each of three schools, and the student with the pharmacy major attended all groups during student presentations.

### Research Design and Development of Surveys

We conducted a mixed-methods study to evaluate the effectiveness of this IPE-IPC program. The quantitative data was collected to understand students' knowledge change before and after the program. The qualitative data was collected to identify students' experiences of attending the program and provide feedback for future programs.

Before the program began, a four-question survey was distributed to all students to assess their baseline knowledge about the association between oral health and overall health (Table 2). At the end of this program, the project team distributed another survey with four closed-ended questions and two open-ended questions. Students were surveyed after the 8-weeks program to gauge the degree of learning and their IPE-IPC experience. At the beginning of each scenario, a five-question survey designed to assess their understanding of each chronic condition was administered. After each scenario, another five-question survey was given to test how much students had learned. Both pre- and post-scenario surveys used the same questions with modifications for each of four conditions: ALZ, OC, PKD, and STK.

**Table 2:** Survey questions.

**I.**	**Pre-program survey**
Q1	Oral health (OH) is an important aspect of overall health
Q2	ER Department and hospitals need dentists on team of physicians
Q3	Physicians or nurses receive enough education on oral health to recognize OH problems
Q4	The OH of the elderly who resides in nursing home facilities or hospice care may be neglected
**II.**	**Post-program survey**
Q1	This project helped me to see things I never paid attention and did not know
Q2	All providers across healthcare must assess oral health condition periodically in the elderly, particularly those in long-term care facilities
Q3	This project helped me to understand that communication is important for better treatment and health outcomes
Q4	I am still puzzled as to how to establish open communications with other professions across disciplines ask unrelated questions to my own area
Q5^*^	Please take a moment and in your words write how this pilot helped you understand the meaning and the value of interprofessional education and interprofessional collaboration
Q6^*^	If you had all the power to change one thing about this issue, what would you do
**III.**	**Pre-scenario survey (applied to four respective chronic conditions)**
Q1	It is necessary to assess oral health condition in victims of *stroke* in rehabilitation and long-term care facilities
Q2	Treating and monitoring patients with *stroke* is irrelevant to oral health condition
Q3	Poor oral health condition is life threating for the elderly with severe physical and cognitive decline
Q4	Oral health is the gateway to overall health and quality of aging
Q5	Periodontal disease and root caries are normal aging process
**IV.**	**Post-scenario survey (applied to four respective chronic conditions)**
Q1	I did not know the extent of the relationship between oral health and overall health condition of the *stroke* patient.
Q2	I will pay more attention to side effects of drugs on *stroke* patients' overall condition including oral condition
Q3	I will include dental history as a part of health history of the *stroke* patients I treat
Q4	I feel uncomfortable to call a professional with questions that I do not know much about
Q5	I recognized how the *stroke* patient's health condition require better integration of interprofessional collaboration to improve the treatment outcomes

* *Open-ended questions*.

All surveys were developed by the research team and the data collection was approved by the Institutional Review Board at University of Texas Health Science Center at Houston. Given a pilot program in one single University, we did not test the reliability and validity of the surveys. We also did not collect demographic information on the 13 students.

### Data Analysis

Students responded to each question using a 5-point Likert Scale from Strongly Disagree (=1) to Strongly Agree (=5). A higher score means a more positive attitude. For the two surveys given before and after each scenario, the responses of the questions with negative wording were reversed from 5 to 1. This was an educational initiative with a small sample size (i.e., 13 students). Therefore, we performed only descriptive analyses presenting the average points and standard errors for each question by each survey. Paired samples Wilcoxon tests were also conducted to compare the scores between pre- and post-scenario surveys. The final assessment included two open-ended questions allowing students to describe how this program helped them understand the value of IPE-IPC and articulate what change they would like to make (Table 2). We synthesized the qualitative responses for these questions and reported the results based on the common themes discovered. The study has been approved by Institutional Review Board at the University of Texas Health Science Center at Houston.

## Results

### Improved Understanding of the Importance of Oral Health

**Figure 1:**
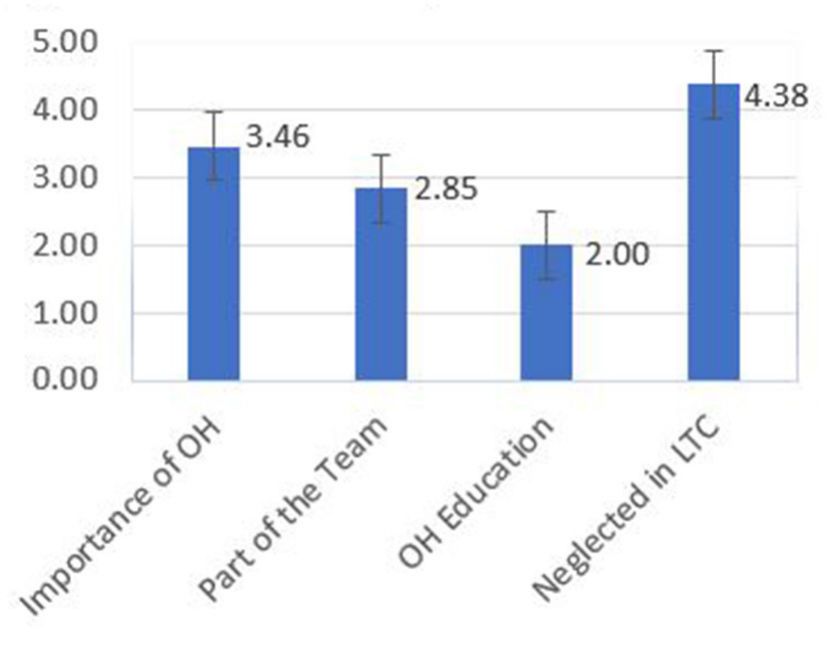
Initial survey results.

Figure 1 shows the results of a four-question survey given to students at the beginning of the educational initiative. The students had a positive belief that oral health is an important aspect of overall health (mean = 3.46), dentists should be a part of the team (mean = 2.85), and that oral health is neglected in nursing home facilities (mean = 4.38). However, the students expressed that they did not get enough education on oral health as a mean value of 2.0 on a 5.0-point Likert score.

**Figure 2:**
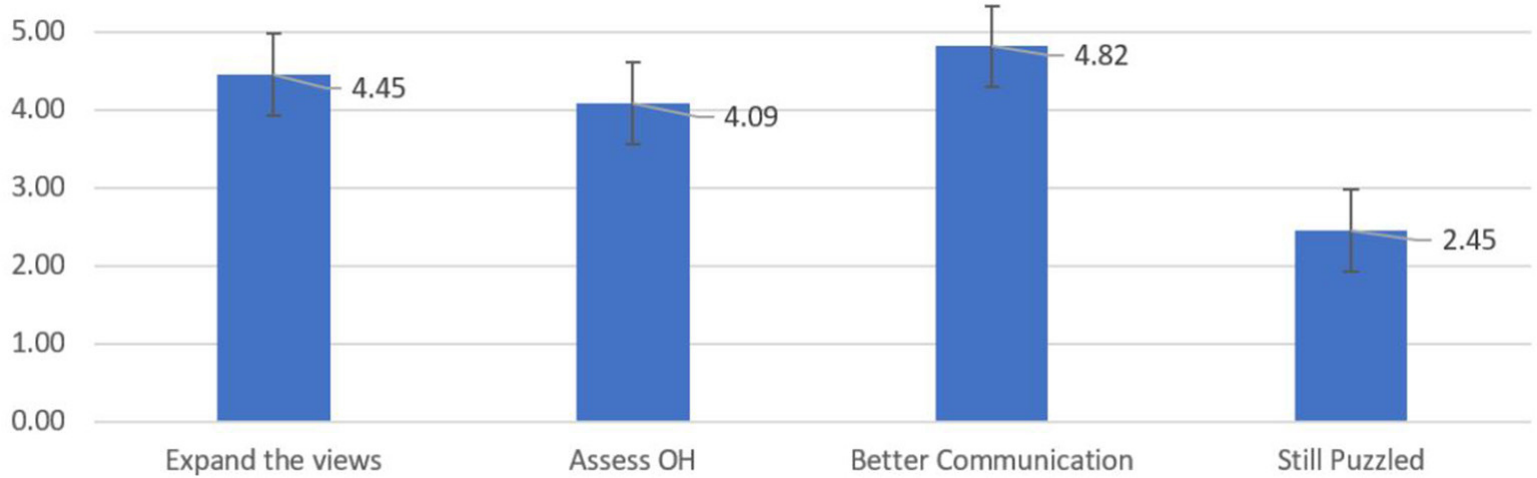
Results of final assessment. Scale from 1 = disagree to 5 = agree.

Figure 2 illustrates the results of another four-question survey given to the students at the end of the educational initiative. After 8 weeks of the IPE-IPC activities, the students were positive that the project helped them to see things they never paid attention to before (mean = 4.45), learn the value of oral health assessment (mean = 4.09), and understand the importance of communication for better treatment and health outcomes (mean = 4.82). Based on the mean value of 2.45 on a 5.0-point Likert score, the students disagreed that they still feel puzzled on how to set up open communications with other professions.

### Enhanced Knowledge to Address Chronic Conditions

Table 3 presents the average score of 13 students for each question in the pre-scenario and post-scenario surveys. Although the content differed between the two surveys, it is obvious that, before the class, students were more likely to disagree with each statement (mean values ~ = 3). After practicing in the case scenario, the overall score became higher (mean values > 4.0). The case on PKD had the most improvement in scores, from 2.72 to 4.43, while the case on STK had the least improvement, from 3.35 to 4.29. With 5.0 being the highest rating, the result illustrates that students have learned the importance of oral health and the association between dental problems and each chronic condition (*p* < 0.05).

**Table 3:** Results of pre- and post-scenarios.

**Mean (std.)**	**Q1**	**Q2**	**Q3**	**Q4**	**Q5**	**Total**
**Alzheimer's disease**						
Pre	3.69 (0.29)	2.15 (0.30)	3.15 (0.44)	2.85 (0.42)	2.46 (0.24)	2.86 (0.20)
Post	4.38 (0.14)	4.38 (0.14)	4.31 (0.24)	4.23 (0.12)	4.38 (0.14)	4.34 (0.08)
**Oral cancer**						
Pre	3.69 (0.31)	1.85 (0.22)	3.62 (0.37)	2.92 (0.42)	2.00 (0.34)	2.82 (0.15)
Post	4.54 (0.14)	4.62 (0.14)	4.85 (0.10)	4.15 (0.22)	4.31 (0.13)	4.49 (0.08)
**Parkinson's disease**						
Pre	3.00 (0.32)	2.08 (0.32)	3.38 (0.31)	3.23 (0.32)	1.92 (0.26)	2.72 (0.17)
Post	4.69 (0.13)	4.23 (0.30)	4.54 (0.14)	4.38 (0.14)	4.31 (0.13)	4.43 (0.08)
**Stroke**						
Pre	2.85 (0.34)	3.15 (0.25)	3.92 (0.24)	3.54 (0.33)	3.31 (0.29)	3.35 (0.11)
Post	4.85 (0.10)	4.69 (0.13)	4.69 (0.13)	4.08 (0.31)	3.15 (0.50)	4.29 (0.16)

*The choices for each question are from Strongly Disagree (=1) to Strongly Agree (=5)*.

*All the comparisons between pre- and post-scenario surveys have passed the biostatistical significance test (p < 0.05)*.

### High Satisfaction About the Program and Feedback for Future Programs

Since participation in the survey was voluntary, two students did not respond to the final assessment survey. For the first open-ended question about how the initiative helped them, six of 11 respondents mentioned that the initiative helped them learn various options for treating their patients. Also, five students mentioned that they learned other professionals' perspectives and how to develop relationships with others. One student reported that the presence of other health professionals in the team made it possible to find answers for clinical questions relating to medications and oral health. In terms of the changes they would like to see, seven of 11 respondents recommended bringing this kind of interprofessional course or seminar to their school. Two students suggested promoting communication across disciplines to eliminate bias and another two students suggested having an oral health-related course in their medical/nursing training.

## Discussion

Based on the analysis results, the program achieved its three learning objectives, including: (1) students recognized what they do not know about oral health of the elderly, which may affect the treatment outcomes; (2) students acknowledged the importance of IPE-IPC and the value of open communication and team accountability; and (3) students were able to create a similar model for their practices in the future.

### Key Features

Two key features have contributed to the success of the program. First, the program adopted an IPE-IPC model engaging 13 students from four disciplines: medicine, nursing, dentistry, and pharmacy. By using the principles of IPE, our study found that students were able to recognize that close collaboration with professionals across disciplines is fundamental to achieving the shared goal of geriatric care (9, 10, 17). In each section, our faculty provided no guidance or solutions to treating the patients. Instead, students collaborated within a group of professionals, deciding together the best course of action to maintain the patient's oral health while treating each chronic condition. With this design, students completed their own tasks based on the training in respective disciplines; meanwhile, they fulfilled their responsibility as a team member. They communicated with different professionals and documented the conversations to ensure their clinical decisions met the patient's preferences. After practicing four cases, non-pharmacy students discovered that pharmacists are the best resource for the patient's medication history and the potential side effects to medications, such as tissue overgrowth or inflammation. Also, engaging pharmacists may resolve the issue of polypharmacy which is commonly seen in geriatric populations.

The second key feature is that the program used four commonly-seen chronic conditions with oral complications in geriatric patients for practice. Consistent with other study findings, students in this program learned how other disciplines evaluate patients and determine treatment plans (10, 16). Four elderly cases were presented with health conditions whose treatment required communication among all providers within the 4Ms framework. For instance, what matters encourages dental students to know and align dental services with what matters to each elderly adult. Mentation reminded students to handle physical illness and alleviate mental sufferings together. Besides, each chronic condition was presented with one oral complication, which further enhanced non-dental students' knowledge about oral health and dental practices, such as ensuring sufficient oral hydration to prevent tooth decay. Integrating the 4Ms model with a focus on oral complications allowed students to gain more confidence in addressing the complex health needs of aging populations. Given the worldwide expansion of aging populations, more IPE-IPC programs that train students to acknowledge the importance of oral health and collaborate across disciplines are highly recommended (1, 7, 17).

### Challenges and Suggestions

While learning was effective based on our study finding, we confronted two challenges that might potentially reduce the benefits of the project. One was the difficulty recruiting students from the College of Pharmacy at the Texas A&M University, partly due to a long driving distance. Because of the existing gap in health culture between oral health and overall health, it was also challenging to recruit students from medical and nursing fields and promote how this project may benefit them (8). Universities tend to emphasize achievement in singular areas of study, but achieving optimal health outcomes requires breaking down the wall between general health and oral health. With our findings, we highly recommend that future healthcare professional programs foster a culture of collaboration and develop more IPE-IPC programs for students and trainees (6, 7, 17, 18).

Another challenge was getting the students to commit to 8 weeks at the end of the school day when they were already overloaded with schoolwork. For faculty members who would like to replicate our program, we highly suggest integrating this IPE-IPC project into the formal curriculum to resolve this significant challenge. Participating in IPE-IPC activities should not be an extracurricular activity that students need to fit it into their schedule (9, 10). Instead, it should have support from institutional leaders who can allocate appropriate resources to ensure the success of the program. It may take the form of an elective course offered by schools of medicine, nursing, dentistry, pharmacy, allied health, and public health. Offering incentives such as a certificate of completion, a letter of acknowledgment, or elective course credits should also be considered to enhance future participation (17, 18).

### Study Limitations

Although this is one of the first program involving four disciplines of students in treating geriatric patients with four chronic conditions, there are some limitation to this pilot project. First, the results could not be generalized to interprofessional programs focusing on different clinical conditions. The program was designed to treat chronic conditions but not infectious diseases or emergencies (e.g., injury). In addition, the design of this IPE-IPC program only allowed for one-time contact, while most chronic conditions require multiple follow-up visits. By seeing the severe outcomes of aging populations during the Covid-19 pandemic, we highly recommend developing programs that expand to a wider scope of practice (20).

Second, we did not test the validity and reliability of the surveys, given that this was a pilot program with 13 students in this cohort. The original design was to recruit 12 students of each discipline in their senior years. Lack of volunteer students and only one pharmacy student representing in all groups might minimize the diversity. It is also unknown if students were already exposed to other similar interprofessional training. Without a control group for comparison, no causation can be determined either as to whether students' improvement in knowledge and attitude was solely based on the participation in our program. Thus, caution should be taken when interpreting the findings of the quantitative data analysis. Yet, through collecting the qualitative data, we were able to evaluate how much students enjoyed the program and whether they acquired the knowledge and skills we expected. We also received valuable feedback on how to enhance the program and suggestions on having IPE-IPC in every school's curriculum. Additional studies with expanded number of students, along with the assignment of a control group, are also recommended to better understand the program's impact on improving students' knowledge and enhancing their clinical experiences.

## Conclusions

Despite the challenges of initiating an IPE-IPC curriculum across four schools, our study found that creating an interactive educational platform can prepare students to continue with a collaborative mindset as health providers and achieve better patient-centered outcomes for all patients. Considering the increasing number of aging populations, it is critical to develop more IPE-IPC programs with the 4Ms framework and a focus on oral complications. In addressing the complex needs of elderly patients, our findings suggest that IPE-IPC should include pharmacists, who make a crucial connection between providers and the treatment outcomes of their patients. It would also be meaningful to evaluate such a program with standardized instruments to supply further evidence on the effects of IPE on students' knowledge and preparedness for clinical practice.

## Data Availability Statement

The raw data supporting the conclusions of this article will be made available by the authors, without undue reservation.

## Ethics Statement

The studies involving human participants were reviewed and approved by IRB (#181206) of the University of Texas Health Science Center at Houston. Written informed consent for participation was not required for this study in accordance with the national legislation and the institutional requirements.

## Author Contributions

In this study, MT was responsible for the overall development and production of the program. W-CL assisted MT with data analysis and manuscript writing. All authors contributed to the article and approved the submitted version.

## Conflict of Interest

The authors declare that the research was conducted in the absence of any commercial or financial relationships that could be construed as a potential conflict of interest.
