# Corrigendum: A Pilot Study of an Interprofessional Program Involving Dental, Medical, Nursing, and Pharmacy Students

**DOI:** 10.3389/fpubh.2020.642239

**Published:** 2021-02-08

**Authors:** Maryam Tabrizi, Wei-Chen Lee

**Affiliations:** ^1^General Practice and Dental Public Health, School of Dentistry, The University of Texas Health Science Center at Houston, Houston, TX, United States; ^2^Office of Health Policy and Legislative Affairs, The University of Texas Medical Branch, Galveston, TX, United States

**Keywords:** oral health, interprofessional education, collaboration, age-friendly, public health

In the original article, we neglected to include the funder **Health Resources and Service Administrator-HRSA**, **grant** # K01HP33459 to **Dr. Tabrizi**. These statements should also be added per funders request.

“This publication was made possible by Grant Number K01HP33459 from the Health Resources and Services Administration (HRSA), an operating division of the U.S. Department of Health and Human Services. Its contents are solely the responsibility of the authors and do not necessarily represent the official views of the Health Resources and Services Administration or the U.S. Department of Health and Human Services.”

The authors apologize for this error and state that this does not change the scientific conclusions of the article in any way. The original article has been updated.

